# The Associations Between Chronotype and Mental Health in Nursing Students

**DOI:** 10.3390/jcm14134440

**Published:** 2025-06-23

**Authors:** Vanessa Ibáñez-del Valle, Rut Navarro-Martínez, Omar Cauli

**Affiliations:** 1 Department of Nursing, Faculty of Nursing and Podiatry, University of Valencia, c/de Méndez y Pelayo, 19, 46010 Valencia, Spain; maria.v.ibanez@uv.es (V.I.-d.V.); omar.cauli@uv.es (O.C.); 2 Frailty and Cognitive Impairment Organized Group (FROG), University of Valencia, 46010 Valencia, Spain; 3 Chair of Active Ageing, University of Valencia, 4610 Valencia, Spain

**Keywords:** chronotype, nurses, students, depression, anxiety, stress

## Abstract

**Background/Objectives**: Numerous studies have documented the effect of human chronotypes on psychological well-being. This study aimed to examine the associations of chronotype subtypes and mental health among Spanish university students. **Methods**: Sociodemographic data were collected electronically using a self-administered questionnaire with Google Forms. In addition, participants completed The Morningness-Eveningness Questionnaire (MEQ) by Horne and Östberg, the Goldberg Anxiety and Depression Scale (GADS), and the Perceived Stress Scale (PSS). **Results**: Two hundred and eighty-nine students participated in the study. The most prevalent chronotype in the study sample was the intermediate (64.4%), followed by the evening (19.4%) and the morning (16.3%) chronotypes. Based on cut-off scores of the instruments used, the percentage of students with relevant symptoms of anxiety, depression and stress was high, 45.3%, 46.4% and 79.6%, respectively. Statistical analysis showed statistically significant differences between the total score of the Goldberg scale and the three chronotype categories, with higher scores in the evening group. A multivariate regression model analysis also identified a statistically significant correlation between the depression subscale and chronotype (R squared = 0.287) and between evening vs. morning chronotype (OR = 0.48; IC 95% [0.23–0.98]) and evening vs. intermediate chronotype (OR = 2.60; 95% CI [1.00–5.08]). In turn, the depression subscale showed a statistically significant correlation with the variables gender (OR = 2.22; 95% CI [1.03–4.76] being more frequent in women) and daily consumption of stimulant drinks (OR = 0.54; 95% CI [0.31–0.94]; being higher in people with lower consumption). The anxiety subscale showed a statistically significant correlation with chronotype (R squared = 0.309) and with evening vs. morning chronotype (OR = 0.46, 95% CI [0.22–0.94]). With respect to stress, there was a statistically significant correlation with gender (OR = 3.08, 95% CI [1.40–6.79], being more frequent in women), with chronotype (R squared = 0.141), and with evening vs. morning chronotype (OR = 0.34, 95% CI [0.16–0.72]). **Conclusions**: Our results suggest that students with an evening chronotype are more likely to suffer from mental health problems, and interventions to improve their mental health are necessary.

## 1. Introduction

Every day, the world changes on a scheduled basis, approximately every 24 h. As a result, living things have had to develop internal systems that prepare them for this change. These systems have allowed different animal species to adapt to the time of daylight or darkness in their environment through rhythms of activity aligned with the daily cycle of light and dark [1,2,3]. In this way, there are biological variables that show a defined periodicity with a cycle length of 24 h. These oscillations of variables are called circadian rhythms and include physiological, psychological, and behavioural variables [3]. Psychological variables include mood, alertness, sleepiness, and task performance [4,5]. For humans, Horne and Östberg [6] identified this variability in sleep pattern (morningness/eveningness preferences), referring to early risers as “morning types” and late risers as “evening types”. These individual differences affecting the timing of sleep and activity during a 24 h day are known as chronotypes [7]. It is an intrinsic characteristic of each human being, which can be classified according to the circadian rhythmic expression of sleep and activity [8]. Studies have shown that the evening chronotype can be a risk factor for depressive disorders and substance use disorders, while the morning chronotype is a protective factor [4,9,10,11].

Some circadian rhythms are misaligned in the university environment relative to the non-university population [12]. This may be due to different social habits of student life: irregular work/rest schedules, preoccupation with exams, relationships with classmates, place of residence/independent living, late nights due to late-night internet surfing, partying, work, alcohol and other drug use and/or abuse, time management, self-regulation, and living with peers [13,14,15]. This may be a risk factor for various health problems, including sleep problems and anxiety [16].

Registered nurses suffer from depression at almost twice the rate of individuals in other professions [17]. On the other hand, college students face challenges related to independent living and academic difficulties. This predisposes them to depression, anxiety and stress, which are standard [18,19]. Many studies on depression among nurses have been mainly related to shift work, which is the primary mode of work for nurses [20,21]. Many health professionals, such as nurses, work at night and must sleep during the day. These work shifts affect their sleep quality and sleep quantity and misalign in the individual’s circadian rhythm [22]. Sleep quality is fundamental to health, and its cumulative effects have been associated with serious health problems. These problems include diabetes, cardiovascular disease, obesity, depression, anxiety, myocardial infarction, and stroke [22,23]. Health problems of nurses and nursing students also affect patients and organisations. Nurses are key to patient safety, as they are directly responsible for patient care [20]. In this regard, it has been shown that depression in nurses can affect both professional performance and lead to errors and negligence [24,25]. Furthermore, depression is linked to increases in work absenteeism, short-term disability, and decreased productivity [17]. The leading causes of drug preparation and administration errors have been shown to include stress, fatigue, increased workload, night shifts, nurse staffing ratios, and workflow interruptions [20].

Prevention is key to avoiding mental health problems. In the case of nurses and nursing students, due to their vulnerability to specific mental health problems such as depression and its impact on patient safety, preventive measures are of particular interest. These measures could be initiated during their university training since university students may undertake shift work experience in hospitals that could hurt their health. Because evening type has been linked to an increased risk of depression, preventive measures could include an assessment of individual chronotype. In this regard, it is helpful to study the chronotype preferences of nurses and nursing students to promote self-care and implement appropriate measures to ensure patient safety. In this way, by preventing mental health problems in nurses, we will also be preventing health problems in the patients under their care and ensuring better functioning of the hospitals and organisations where they work, with lower absenteeism and improved productivity. In addition, implementing self-care behaviours to prevent mental health problems in nursing students can benefit them as they transition from undergraduate to graduate nursing roles [26].

Studies have been conducted on the influence of chronotype on university students’ academic performance and learning motivation outcomes [27,28]. The impact of chronotype on social jet lag and quality of life has also been examined [29]. However, regarding university students, there are few studies linking chronotype to mental health issues such as depression, anxiety and stress. A study involving Chinese medical students revealed a significant association between chronotype and depressive symptoms [30]. Chang et al. [31] found that an evening chronotype among medical students posed a risk factor for anxiety and depression. Another study involving college students indicated that being an early riser might serve as a protective factor, not only against depression but also against suicide [32]. As far as studies analysing the relationship between chronotype and mental health in undergraduate nursing students in Spain are concerned, none exist in the scientific literature. Thus, to our knowledge, this is the first study to analyse the relationship between chronotype and depression, anxiety, and stress in university nursing students in Spain. On the other hand, in addition to analysing the relationship between chronotype and mental health in nursing students, it also studies for the first time the role played by sociodemographic factors in this association.

## 2. Materials and Methods

### 2.1. Study Design and Population

A cross-sectional study was conducted between October 2022 and March 2024 with university students enrolled in the Nursing degree at the University of Valencia (Valencia, Spain). The study was carried out in accordance with the requirements of the Declaration of Helsinki and was approved by the Research Ethics Committee of the University of Valencia (protocol number 2298864, 13 October 2022). Before accessing the data collection instrument, participants received detailed information about the purpose of the study and provided their consent by agreeing to complete the questionnaire.

The sample was obtained through non-probabilistic convenience sampling, selecting participants based on their accessibility. The only exclusion criterion was refusal to participate. A random sample of 293 individuals was determined to be sufficient to estimate, with a 95% confidence level and a precision of ±5 percentage points, a population percentage expected to be around 45% [33]. A follow-up loss rate of 10% was estimated. The sample size was calculated using the Poisson approximation.

### 2.2. Data Collection

An anonymous, self-administered questionnaire was created using Google Forms and distributed electronically for data collection. The link to access the survey was sent through the Official Teaching Support Resources Platform (Virtual Classroom) of the Universitat de València. Participants were recruited October 2022 and March 2024; during this period, reminders were sent to encourage their participation.

The questionnaire collected sociodemographic, academic, lifestyle, and clinical data. Sociodemographic variables included age, sex, marital status, or existence of a stable partner, whether they had children, cohabitation situation, and employment status. Similarly, other previous population-based studies on mental health, such as You et al. (2024), used similar socio-demographic variables [34]. Regarding lifestyle, questions were asked about the consumption and frequency of stimulating beverages (coffee, tea, or cola), tobacco use, alcohol consumption, and psychoactive substances. Alcohol consumption was assessed using the AUDIT-C3. Participants also indicated their weight (kg) and height (cm) for BMI calculation, whether they had any chronic illness, and if so, which one, as well as their self-perception of overall health status using a visual scale from 1 to 10, where a higher score corresponded to a better self-perception of health status. Additionally, we used the Morningness-Eveningness Questionnaire (MEQ) by Horne and Ostberg [6] to assess chronotypes, the Goldberg Anxiety and Depression Scale (GADS) [35] to evaluate symptoms of anxiety and depression, the Perceived Stress Scale (PSS) [36] to assess stress levels, and the short form of the IPAQ [37] to evaluate activity levels.

### 2.3. Chronotype Assessment

The Morningness-Eveningness Questionnaire (MEQ) by Horne and Ostberg was used to assess the individual’s chronotype based on 19 multiple-choice questions that evaluate the individual’s preferred timing for a variety of daily activities. The total score ranges from 16 to 86 points. A score above 58 indicates a morning chronotype, while a score below 42 suggests an evening chronotype. Scores between 42 and 58 are classified as intermediate chronotype [6].

### 2.4. Assessment of Depressive and Anxiety Symptoms

The presence of anxiety and/or depression symptoms was assessed using the Spanish version [38] of the Goldberg Anxiety and Depression Scale (GADS) [35]. This scale consists of two subscales, one for anxiety and one for depression, each with 9 items with dichotomous responses (YES or NO). Each time a participant answers “yes” to a question, they receive one point. On the anxiety subscale, 4 or more affirmative responses indicate the presence of anxiety symptoms, while on the depression subscale, 2 or more affirmative responses are compatible with depressive symptoms. On both subscales, the higher the number of affirmative responses, the greater the severity of the problem, with a maximum score of 9 points for each.

### 2.5. Assessment of Self-Perceived Stress

The Perceived Stress Scale (PSS) is a tool that assesses individuals’ perception of stress through a 10-item questionnaire [36]. Participants respond to questions about how often they have experienced everyday situations as stressful in the last month, using a response scale ranging from “never” (a score of 0) to “very often” (a score of 4). Scores are summed to obtain a total that ranges from 0 to 40 points, where a higher score indicates a greater level of perceived stress. In the same way as in previous studies [39,40], Perceived Stress Scale (PSS) scores were categorised into quartiles, making it easier to analyse and compare groups. Thus, a cut-off of 21 or more was established for high stress. The Spanish translation of the scale, validated in previous research, was used for this study [41].

### 2.6. Level of Physical Activity

Levels of physical activity were assessed using the short form of the IPAQ [37], a self-reported questionnaire consisting of seven questions designed to evaluate the frequency and duration of vigorous activity, moderate-intensity activity, and walking over the last week, as well as the time spent sitting each day. This questionnaire has been validated in Spanish [42]. Scores were calculated in terms of MET-minutes/week for each type of activity, allowing for a comprehensive assessment of total physical activity performed. Overall levels of physical activity were categorised as low, moderate, or high based on self-reports from the IPAQ.

### 2.7. Statistical Analysis

Descriptive analysis of the quantitative variables was performed by calculating measures of central tendency and dispersion, such as the mean, standard error of the mean (SEM), and ranges. The normality of the distribution of each variable was assessed using the Kolmogorov–Smirnov test. For comparison of means between two independent groups, the Mann–Whitney U test was used, while for comparison of means between three or more independent groups, the Kruskal–Wallis test was employed. Differences in proportions were analysed using the Chi-square test. Associations between quantitative variables were explored using Spearman’s rank correlation coefficient.

Logistic regression was performed to try to determine which variables were related to clinically significant depressive symptoms, anxiety and stress level by making a predictive model of these symptoms including variables such as age, gender, weekly physical activity, consumption of alcohol, stimulant drink, smoking habits and body-mass index. This technique can be used to simultaneously assess several factors presumed but necessarily related to the dependent variable. Thus, we obtained measurements (odds ratios) of the association between each variable adjusted to all the other variables to detect possible interactions between them and the effect studied. The regression model was repeated by using the depressive score dichotomized by the Goldberg scale cut-off for depression subscale (≥2) as the dependent variable for the anxiety subscale (≥4). For the stress scale, no cut-off is available, and the scores were dichotomized based on high quartile score (means high stress level) versus the other 3 quartiles pooled together. Statistical significance was set at *p* < 0.05. The statistical analysis was performed using the SPSS 28.0 software package (SPSS Inc., Chicago, IL, USA).

## 3. Results

### 3.1. Characteristics of the Sample

The sample comprised 289 nursing degree students, 86.5% women (*n* = 250) and 13.5% (*n* = 39) men, with an average age of 20.60 (±2.56) years (range = 17–30 years), who completed the online survey. Further, 95.5% of the students were single (*n* = 276), 99.7% were childless (*n* = 288), and 69.5% lived mainly in their nuclear family (*n* = 198). Likewise, 82.4% (*n* = 238) of the students reported not suffering from any chronic disease, 74% (n = 214) had a normal weight, according to their BMI (kg/m^2^), and, with an average score of 7. 79 (±1.29) points out of 10 points, showed a good self-perception of their state of health. A total of 13 students reported using psychotropic drugs daily.

### 3.2. Consumption of Coffee, Cola, Tea, Tobacco, and Alcohol

A total of 61.9% of the students (*n* = 179) reported consuming stimulant drinks daily, with an average number of cups of coffee consumed per day of 1.81 (±0.91), 1.47 (±0.73) cups of cola, and 1.29 (±0.50) cups of tea. While 92% (*n* = 266) reported not smoking, 58.1% (*n* = 121) reported alcohol consumption. Of the students who consumed alcohol, more than half (*n* = 98) did so between 2 and 4 times a month. The amount consumed per occasion was at least 3–4 drinks in a third of cases (*n* = 32), with 3% (*n* = 3) consuming more than five drinks. In addition, 1.3% (*n* = 4) of the total number of students surveyed reported using psychoactive substances, among which they indicated cannabis and marijuana.

### 3.3. Chronotype

Most students had an intermediate chronotype, with 64.4% (*n* = 186) of students having this type. The second most frequent chronotype was the evening chronotype, with 19.4% (*n* = 56) of the students. Finally, 16.3% (*n* = 47) of the students were of the morning chronotype.

### 3.4. Physical Activity

Physical activity was measured using the IPAC questionnaire, and activity levels were classified into three categories: low, moderate, and high. Of the participants studied, 15 had an activity level corresponding to low or inactive (5.2%), while 92 (31.8%) had moderate physical activity levels. The rest were counted as missing values. The IPAC scale records weekly activity in Mets (Metabolic Equivalent of Task or Metabolic Index Units) per minute per week. The mean number of Mets in the sample studied was 2574.60 (±2780.708). There were no significant differences between the number of Mets performed by men and women. There were also no significant differences between the number of Mets and the chronotype categories. Regarding the mental health of the participants, there were no significant differences between the number of Mets performed and the scores on the Goldberg and Self-Perceived Stress scales.

### 3.5. Mental Health: Anxiety, Depression, and Stress

#### 3.5.1. Anxiety and Depressive Symptoms

On the Goldberg scale, the cut-off points are four or more for the anxiety subscale and two or more for the depression subscale, with higher scores indicating a more severe problem (the maximum possible being 9 for each subscale) and the maximum in both scales is 18. The overall mean score of the scale was 4.46 (±2.320). On the anxiety subscale, the mean was 2,83 (±1.345); on the depression subscale, the mean was 1.63 (±1.357). Considering a cut-off point on the anxiety subscale of four points or more, 45.3% (n = 131) of the participants are at risk of suffering from anxiety. For depression, and considering a cut-off point of two or more points, 46.4% (n = 134) are at risk of suffering depression.

Regarding the difference in the results of the subscales according to gender, the mean score of the anxiety scale in men was 2.44 (±1.51), while in women, it was 2.89 (±1.30). Regarding the depression subscale, the mean depression score in men was 1.23 (±1.18), while in women, it was 1.70 (±1.37). While gender did not show significant differences with the anxiety subscale (*p* = 0.104), we observed a trend towards depression in favour of women (*p* = 0.05).

In terms of age, students with higher anxiety symptoms (anxiety subscale score ≥ 4 points) had a significantly greater mean age (20.84 (±2.46) years) than students with a score on the anxiety subscale < 2 points (20.41 (±2.65) years, *p* = 0.039). There were no significant differences between the age of the students and the symptoms of depression (*p* = 0.157). We did not observe significant differences between the mood of students with marital status, having children and place of residence during the academic year, smoking, and consumption of alcohol or stimulant drinks (*p* > 0.05, in all cases). No relationship was observed between daily coffee consumption and the development of depressive symptoms (*p* = 0.292), nor with anxiety symptoms (*p* = 0.273). Likewise, we observed that those students with more severe symptoms of anxiety and/or depression reported a worse self-perception of their health status (*p* < 0.001; for all scales). Finally, while the consumption of psychotropic drugs daily was not associated with symptoms of anxiety (*p* = 0.301), students who reported daily use of psychotropic drugs, compared to those who did not consume, had significantly higher mean scores on the Goldberg total scale (5.85 (±2.30) versus 4.40 (±2.30)) and the depression subscale (2.69 (±1.25) versus 1.58 (±1.34)) (*p* = 0.037 and *p* = 0.005, respectively).

#### 3.5.2. Self-Perceived Stress

The mean score on the Perceived Stress Scale was 23.54 (±4.115). Men scored on average 22.18 (±3.02) and women 23.76 (±4.22), finding statistically significant differences between men and women (*p* = 0.002). Considering a cut-off point of 21 (third quartile), 79.6% of the participants reported stress symptoms.

### 3.6. Chronotype and Its Relationship with Sociodemographic Characteristics, Lifestyle, and Mental Health Conditions

No significant differences were observed between chronotype type and gender (*p* = 0.459), marital status (*p* = 0.939), having children (*p* = 0.644), and place of residence during the academic year (*p* = 0.377). Age distribution was the same across the chronotype categories (*p* = 0.184). Chronotype was not significantly associated with the consumption of alcoholic beverages (*p* = 0.145). Chronotype was also not significantly associated with stimulant drinks (*p* = 0.723) or their consumption frequency (*p* = 0.071 for coffee, tea, and cola). There was also no association between chronotype and smoking (*p* = 0.492). Chronotype was also not associated with BMI categories (*p* = 0.091), the presence of chronic disease (*p* = 0.073), or daily consumption of psychotropic drugs (*p* = 0.462).

**Figure 1:**
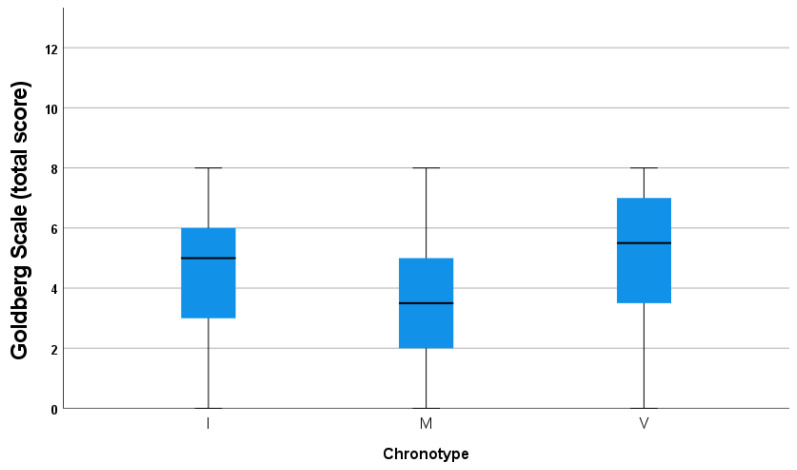
Correlation analyses between Goldberg scale (total score) and chronotype.

Concerning mood symptomatology, there were statistically significant differences between the total score of the Goldberg scale and the three chronotype categories (*p* ≤ 0.01), with higher scores in the evening group. The mean score of the Goldberg scale for the morning and intermediate chronotypes was 3.50 (±2.297) and 4.47 (±2.250), respectively. However, the mean Goldberg scale score in the evening chronotype was 5.23 (±2.31) (see Figure 1).

**Figure 2:**
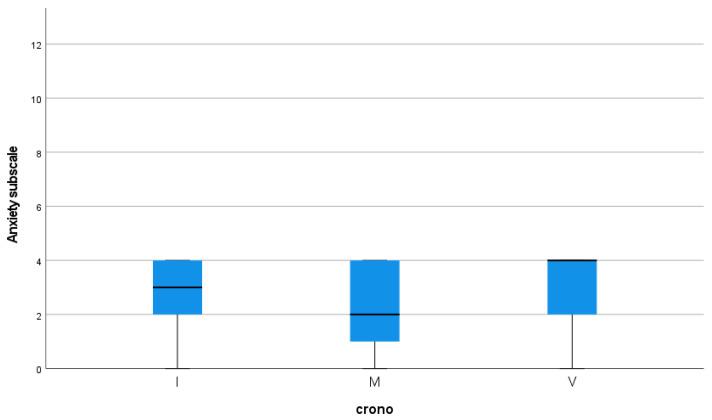
Correlation analyses between anxiety subscale and chronotype.

Regarding the anxiety subscale score, the statistical analysis showed significant differences in the three chronotype categories (*p* = 0.023), with higher scores in the intermediate and evening chronotypes. The mean score of the anxiety subscale was 2.91 (±1.531) in the evening chronotype, 2.93 (±1.288) in the intermediate chronotype, and 2.35 (±1.386) in the morning chronotype (see Figure 2).

**Figure 3:**
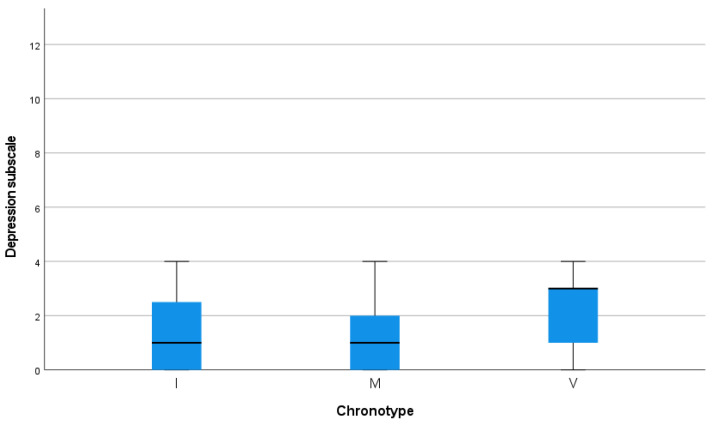
Correlation analyses between depression subscale and chronotype.

Regarding the depression subscale, there were statistically significant differences in the three chronotype categories (*p* ≤ 0.01). The evening chronotype presented higher values than the rest of the chronotype categories; the mean depression subscale score was 2.32 (±1.323), while the mean scores of the intermediate chronotype and the morning chronotype were 1.55 (±1.321) and 1.15 (±1.251), respectively (see Figure 3).

**Figure 4:**
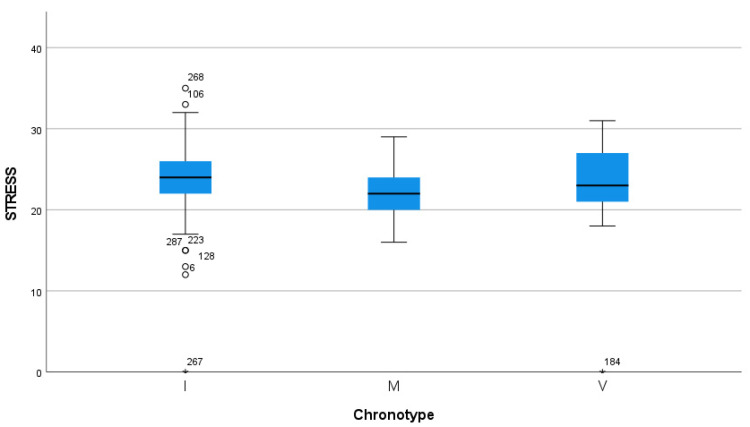
Correlation analyses between self-perceived stress and chronotype.

Concerning the self-perceived stress scale, the analysis showed significant differences between the total scale score and the three chronotype categories (*p* ≤ 0.01), with the intermediate and evening chronotypes having higher scores. In the morning chronotype, the mean score of the self-perceived stress scale was 22.34 (±3.577), while the mean scores of the intermediate and evening chronotypes were 23.85 (±3.984) and 23.52 (±4.798), respectively (see Figure 4).

### 3.7. Symptoms of Anxiety and Depression and Their Relationship to Sociodemographic, Lifestyle, and Health Conditions

As for the mood of university students, the average score of the Goldberg total scale was 4.46 (±2.32) points (range = 0–8 points), with mean scores for the anxiety and depression subscales of 2.83 (±1.34) points (range = 0–4 points) and 1.64 (±1.35) points (range = 0–4 points), respectively. Establishing a cut-off point of 4 points or more in the anxiety subscale and two or more in the depression subscale, it was observed that 45.3% (n = 131) of the students presented symptoms of anxiety and 46.4% (n = 134) of depression. For the total score of the scale, if we consider a value of 6 or more points, which is equivalent to the third quartile, 33.6% (n = 97) of participants showed symptoms of anxiety and depression. There was a statistically significant difference between the total score of the scale with gender, with the average of symptoms of anxiety and depression being higher among women than among men (4.59 (±0.52) versus 3.67 (±2.32); *p* = 0.017). As for the subscales, while gender did not show significant differences with the anxiety subscale (*p* = 0.104), we observed a trend towards depression in favour of women (*p* = 0.05). Regarding age, students with higher anxiety symptoms (anxiety subscale score ≥ 2 points) had a significantly higher mean age (20.84 (±2.46) years) than students with a score on the anxiety subscale < 2 points (20.41 (±2.65) years, *p* = 0.039). There were no significant differences between the age of the students and the symptoms of depression (*p* = 0.399). Nor did they observe significant differences between the mood of students with marital status, having children and place of residence during the academic year, smoking, and consumption of alcohol or stimulant drinks (*p* > 0.05, in all cases). However, we found that students with a higher daily consumption of coffee presented more severe symptoms of depression (*p* = 0.041) but not anxiety (*p* = 0.307). Likewise, we observed that those students with more severe symptoms of anxiety and/or depression reported a worse self-perception of their health status (*p* < 0.001; for all scales) (Figure 2). Finally, while the consumption of psychotropic drugs daily was not associated with symptoms of anxiety (*p* = 0.301), students who reported daily use of psychotropic drugs, compared to those who did not consume, had significantly higher mean scores on the Goldberg total scale (5.85 (±2.30) versus 4.40 (±2.30)) and the depression subscale (2.69 (±1.25) versus 1.58 (±1.34)) (*p* = 0.037 and *p* = 0.005, respectively).

### 3.8. Mental Health Variables Associated with Chronotype Type: Linear Regression Analyses

Multivariate logistic regressions were performed to determine whether significant depressive symptoms, anxiety and high stress level were associated with the type of chronotype and other variables in the multivariate analysis. The dependent variable selected was the score on each mental health symptom scale (stress, anxiety subscale, and depression subscale). Gender, age, chronotype, and level of physical exercise were selected as independent variables. In this way, linear regressions were performed with each of the scores of the mental health scales that were not included as the dependent variable (Table 1). In the multivariate analysis, the anxiety subscale showed a statistically significant correlation only with chronotype (*p* = 0.042) and specifically with evening versus morning chronotype (*p* = 0.034).

The strength of the relationship between the predictors and the depressive symptoms as the outcome was R squared = 0.287 and adjusted R squared = 0.271; anxiety symptoms as the outcome gave R squared = 0.309 and adjusted R squared = 0.294; and for stress symptoms as the outcome, we found R squared = 0.141 and adjusted R squared = 0.123.

Non-standardised coefficients, standardised coefficients, significances, and confidence intervals are presented in Table 1.

## 4. Discussion

To our knowledge, this is the first study conducted to examine the association between chronotype and mental health problems in Spanish university nursing students. Our results show statistically significant associations between the total Goldberg scale score and the depression and anxiety subscales, with higher scores observed in the group of students characterised by the evening-type chronotype. Similarly, a study performed in Chinese medical students [30] reported a positive correlation between evening chronotype and depressive symptoms. Likewise, the study by Park et al. [32] conducted with Korean college students showed that depressive symptoms and suicidality were statistically higher in evening-type chronotype college students than in morning-type college students. These results could be related not only to sleep pattern but also to other variables such as sleep duration or sleep quality. Previous research has shown that, compared to morning people, individuals with a nocturnal chronotype have a longer sleep latency [43] and a shorter sleep duration [10,44]. In addition, studies such as Giannotti et al., 2002, have shown that individuals with nocturnal chronotype have an irregular sleep–wake schedule, poor sleep quality and, greater daytime sleepiness than morning types [45].

Greater emotional intelligence could help regulate mood regardless of chronotype (morning or evening). People with lower emotional intelligence, especially evening types, may be more prone to experiencing variations in their mood throughout the day, which could partly explain the observed association [46]. According to the results of the regression analysis in this study, some socio-demographic variables, such as gender or other lifestyle-related variables, such as daily consumption of stimulant drinks (coffee, cola or tea), were found to be predictors of mental health. Thus, we found statistically significant correlations of the Goldberg depression subscale with both gender and daily consumption of stimulant drinks. In our study, women had more depressive symptoms than men, and people who consumed stimulant drinks daily had fewer depressive symptoms than those who did not consume these substances. Our results are supported by other studies of university students, which have shown that depressive symptoms were higher among women than among men [47,48]. On the other hand, the short-term effects of caffeine consumption on mental health have been documented and include improved mood and alertness [49,50,51]. Previous studies in high school students [52] found that the incidence of depression decreased as the level of caffeine consumption increased. Similarly, caffeine consumption was associated with a lower risk of depression compared to no consumption [21].

Regarding the anxiety subscale score, the results of our study showed significant differences in the three chronotype categories, with higher scores in the intermediate and evening chronotypes. Previous studies have shown results that go in the same direction. Luz et al. [53] conducted a study with Brazilian university health science students and found that morning chronotype students showed lower anxiety levels (state and trait), and the evening chronotype in University Brazilian students was associated with higher anxiety states than the morning type [54].

Regarding the self-perceived stress scale, the analysis of our study shows significant differences between the total score of the scale and the three chronotype categories, with students from the intermediate and evening chronotypes obtaining the highest scores of stress. In a previous study conducted with students from the psychology field [55], a tendency for higher stress levels was reported for the students belonging to the evening chronotype. To our knowledge, the relationship between chronotype and self-perceived stress has not been studied in undergraduate nursing students. This relationship was studied in a recent study conducted in professional nurses, and the results were like those observed in nursing students in the present study; as a matter of fact, the perceived stress scores of the evening chronotype nurses were higher than those of the morning type [56]. According to the results of the regression analysis in this study, gender was found to be a predictor of stress. Thus, women showed higher stress values than men. In general, gender studies in the scientific literature have found that women have higher stress levels than men [57,58], as we have found in our study. Similarly, a recent study of nursing students found statistically significant gender differences in both clinical and academic settings, with women having significantly higher levels of total perceived stress than men [59]. When analysing the effects size in multivariate analyses in order to measure the strength of the relationship between the predictors and the outcome variables, e.g., psychological variables, indicating how much variance of the outcome variable is explained by the model, we found that the most strong were for anxiety symptoms (R squared 0.309) and for depressive symptoms (R squared 0.287), suggesting that around 28–30% of the variance can be explained by the independent variables.

Although most of the students in our study had an intermediate chronotype, the second most frequent chronotype turned out to be the evening chronotype. While chronotype can be modified with light exposure [60], the tendency toward the evening chronotype could be due to environmental demands [61], particularly social demands, which can generate a mismatch known as social jet lag [62]. Social jet lag negatively affects academic performance, particularly in women, by impacting various cognitive abilities and lowering their grade point average [63]. Social jet lag refers to the circadian mismatch that occurs when people shift their sleep schedules from weekdays to weekends [64]. Thus, people with social jet lag tend to sleep too little during the workweek and sleep too much on weekends [65]. Most people suffer from social jet lag throughout their school and working lives, but according to Roenneberg et al. [60], this phenomenon is greatest around the age of 20. This is because the chronotype is, on average, later in adolescence than at any other age. This statement would justify the tendency towards the evening chronotype in our study, since the mean age of our sample was 20.60 years (±2.56). Furthermore, in the case of university students, it could also be influenced by academic activity beginning early in the morning. This schedule would cause students with later chronotypes to have higher perceived stress [66], potentially due to a greater decrease in mood and lower alertness experienced in the morning [67]. This could be especially influential in the case of nursing students who must perform internships in clinical settings at early stages, and in those who must demonstrate high performance in the care of real patients, who can face serious health problems. In our study, no significant differences were observed between chronotype type and other sociodemographic variables such as age, gender, marital status, having children and place of residence during the academic year, smoking, consumption of alcoholic beverages or stimulants, BMI, chronic diseases, or daily consumption of psychotropic drugs. There were also no significant differences between physical activity and chronotype. In this study, a high prevalence of mental health problems (46.4.3% depression, 45.3% anxiety, and 79.6% stress) was found in undergraduate nursing students. In recent years, an increase in the rates of college students experiencing symptoms of depression and anxiety has been observed [68,69]. In a study based on the World Health Organization (WHO) global surveys of university student mental health, involving universities in eight countries (Australia, Belgium, Germany, Mexico, Northern Ireland, South Africa, Spain, United States), approximately 31% of university students experienced some mental health disorder over the course of the past year [70]. Of all mental health problems analysed, the most common was major depressive disorder (21.2%), followed by generalised anxiety disorder. Regarding health sciences students, some studies suggest that the prevalence of mental disorders is higher in students from health sciences compared to other areas of studies [71]. A multicentre study carried out in 43 medical schools in Spain reported an overall prevalence of depression of 41% and of anxiety 25% [71]. In our study, the prevalences of depression and anxiety were even higher, being a little bit higher, e.g., 46 and 45% approx., respectively. This may be because the stressors faced by nursing students differ from those of other disciplines, due in part to the fact that nursing curricula simultaneously involve studying in didactic and clinical settings [72]. Although undergraduate nursing education equips students with the knowledge and skills for practice, facing real situations of patient suffering may increase the risk of students experiencing stress, anxiety, or depression [73]. On the other hand, most participants in our study were female (86.5%) and, therefore, this high prevalence of depressive symptomatology could be because depression is usually higher in women than in men [74,75]. Furthermore, although gender did not show significant differences with the anxiety subscale in our study, a tendency towards depression in favour of women was observed (*p* = 0.05). Regarding age, the students in our study with higher anxiety symptoms had a significantly older mean age (20.84 (±2.46) years) than students with lower scores (*p* = 0.039). This increase in anxiety in older students could be due to being upperclassmen performing special service placements and facing other stressors. In this direction, Yi et al. [76] demonstrated in their study that undergraduate nursing students were more prone to anxiety during the last period of their clinical internship. In addition, these students presented specific sources of anxiety such as career decision making, job search, graduation and licensure examination [77,78,79]. In addition, we detected that the students with a greater burden of symptoms of anxiety and/or depression reported a worse self-perception of their health status according to the findings of another study conducted among Bangladeshi university students [80]. Regarding self-perceived stress, 79.6% of the participants in our study reported stress symptoms. On the other hand, the results of the multivariate regression analysis showed that the chronotype was a variable that predicted self-perceived stress. Thus, there was a statistically significant correlation between stress and evening vs. morning chronotype, with participants with evening chronotype having higher levels of stress. Stress has been identified as a predictor of anxiety among nursing students [78]. High levels of stress could have serious mental health consequences, such as increased risk of anxiety and depression [81,82].

The present study adds new evidence for the association between chronotype and depressive and anxiety symptoms, with students exhibiting an evening-type chronotype displaying a higher burden of these symptoms. This represents a potential subgroup of patients who could benefit from interventions aimed at improving their mental health, as demonstrated by recent studies in various countries indicating that the quality of mental health among young people or young adults is quite low [83]. However, some limitations have been identified in the study that need to be considered when designing future research in this area. Although the majority of our study population was female, it is a representative sample of the population studied. Nursing is a feminised profession, both in terms of the number of university students and the number of registered professionals. According to a recent Ministry report, in Spain, approximately 85.5% of registered nurses are women [84]. However, we cannot generalise our results to university students from other degrees that are not predominantly female, and this is a limitation. Because the information on anxiety, depression, and stress symptomatology was obtained through self-reported scales, we cannot provide a clinical diagnosis to explain the associations observed. Additionally, the sample was a convenience sample, as students opted to participate in the survey voluntarily; thus, we cannot generalise these findings to the entire population. Moreover, in these circumstances, people with mental health problems in the sample studied might have been more likely to participate. This circumstance could have contributed to a bias in the responses. However, the fact that the survey was anonymous may have encouraged participation from students with known mental health problems. Supporting this assumption, the rates of depressive and anxiety symptoms we detected were like those published by other authors. Despite these limitations, our study incorporates validated scales commonly used in clinical settings and research.

## 5. Conclusions

The results of the current study are significant, as they indicate that the morning chronotype serves as a protective factor against mental health issues among university nursing students. Conversely, the evening chronotype is a risk factor, particularly affecting women’s mood. These findings underscore the importance of evaluating chronotype in nursing students and have public health implications, given that chronotype (and gender, in the case of depression) is closely linked to the symptomatology of depression, anxiety, and stress. Although gender and chronotype are non-modifiable individual factors, it is essential to consider the circadian schedule delays that college students may adopt to fulfil social demands, as a potentially modifiable factor. The results obtained in this study could be taken into account in the design of preventive mental health programmes in the university academic setting. Among other measures, it could be interesting to adapt the shifts to the individual chronotypes of the students during the internship rotations in the hospital. Shift work may also be unavoidable for nurses; however, understanding the predictive and chronotype-related factors for nurses is crucial for nurse educators and managers in developing a reasonable shift system and strategies to help nurses and undergraduate students adjust their work to their individual chronotypes. Such measures will enhance student nurses’ mental health, work productivity during internships, and patient safety. Learning to care should begin with self-awareness and self-care. In this respect, prevention and treatment programmes for undergraduate mental health issues should carefully consider these factors, especially concerning young adult women and student nurses.

## Figures and Tables

**Table 1:** Multivariate regression analysis.

Depression								
	B	Standard Error	Wald	gl	Sig.	Exp(B)	95% C.I. for Exp(B)	
							Lower Limit	Upper Limit
Age (years)	−0.081	0.052	2.407	1	0.121	0.922	0.833	1.022
Gender	0.797	0.390	4.185	1	0.041	2.220	1.034	4.764
Daily consumption of stimulant drinks	−0.602	0.276	4.759	1	0.029	0.548	0.319	0.941
Smoking habit	0.568	0.485	1.370	1	0.242	1.765	0.682	4.569
Alcohol consumption	0.006	0.269	0.001	1	0.982	1.006	0.594	1.706
Weekly physical activity (METS)	0.000	0.000	1.355	1	0.244	1.000	1.000	1.000
BMI			2.705	3	0.439			
Underweight vs. normal weight	0.196	0.429	0.210	1	0.647	1.217	0.525	2.820
Underweight vs. overweight weight	0.561	0.559	1.007	1	0.316	1.753	0.586	5.249
Underweight vs. obesity	1.182	0.847	1.949	1	0.163	3.261	0.620	17.146
Chronotype			14.565	2	0.001			
Evening vs. morning	−0.732	0.364	4.044	1	0.044	0.481	0.236	0.982
Evening vs. intermediate	0.955	0.342	7.801	1	0.005	2.598	1	5.076
**Anxiety**								
	**B**	**Standard Error**	**Wald**	**gl**	**Sig.**	**Exp(B)**	**95% C.I. for Exp(B)**	
							**Lower Limit**	**Upper Limit**
Age (years)	0.042	0.050	0.701	1	0.402	1.043	0.945	1.150
Gender	0.332	0.375	0.783	1	0.376	1.394	0.668	2.907
Daily consumption of stimulant drinks	0.268	0.271	0.976	1	0.323	1.307	0.768	2.224
Smoking habit	0.366	0.481	0.578	1	0.447	1.441	0.561	3.701
Alcohol consumption	−0.153	0.266	0.330	1	0.565	0.858	0.510	1.445
Weekly physical activity (METS)	0.000	0.000	2.180	1	0.140	1.000	1.000	1.000
BMI			4.746	3	0.191			
Underweight vs. normal weight	−0.595	0.418	2.031	1	0.154	0.551	0.243	1.250
Underweight vs. overweight weight	0.124	0.552	0.050	1	0.822	1.132	0.384	3.338
Underweight vs. obesity	0.011	0.785	0.000	1	0.989	1.011	0.217	4.710
Chronotype			6.331	2	0.042			
Evening vs. morning	−0.774	0.365	4.485	1	0.034	0.461	0.225	0.944
Evening vs. intermediate	0.291	0.324	0.804	1	0.370	1.337	0.708	2.524
**Stress**								
	**B**	**Standard Error**	**Wald**	**gl**	**Sig.**	**Exp(B)**	**95% C.I. for Exp(B)**	
							**Lower Limit**	**Upper Limit**
Age (years)	0.034	0.063	0.298	1	0.585	1.035	0.915	1.170
Gender	1.125	0.403	7.793	1	0.005	3.081	1.398	6.790
Daily consumption of stimulant drinks	0.044	0.335	0.017	1	0.896	1.045	0.542	2.013
Smoking habit	−0.406	0.575	0.497	1	0.481	0.667	0.216	2.058
Alcohol consumption	0.274	0.327	0.699	1	0.403	1.315	0.692	2.498
Weekly physical activity (METS)	0.000	0.000	0.354	1	0.552	1.000	1.000	1.000
BMI			2.174	3	0.537			
Underweight vs. normal weight	−0.526	0.592	0.790	1	0.374	0.591	0.185	1.885
Underweight vs. overweight weight	−0.581	0.720	0.652	1	0.420	0.559	0.136	2.294
Underweight vs. obesity	−1.290	0.878	2.157	1	0.142	0.275	0.049	1.539
Chronotype			7.980	2	0.018			
Evening vs. morning	−1.083	0.383	7.979	1	0.005	0.338	0.160	0.718
Evening vs. intermediate	−0.336	0.402	0.699	1	0.403	0.715	0.325	1.570

## Data Availability

The data presented in this study are available on request for scientific purposes from the corresponding author.
